# Supplementary material for: Human Umbilical Cord Lining-Derived Epithelial Cells: A Potential Source of Non-Native Epithelial Cells That Accelerate Healing in a Porcine Cutaneous Wound Model
Source: Int J Mol Sci. 2022 Aug 10;23(16):8918. doi: 10.3390/ijms23168918 (PMC9408523; doi:10.3390/ijms23168918)
Supplement: Supplementary file 1 [file ijms-23-08918-s001.zip › ijms-1819350-supplementary.pdf]

Human umbilical cord lining-derived epithelial cells:

A potential source of non-native epithelial cells that accelerate healing in a porcine cutaneous wound model

Kua et al.

Supplementary Figures:

a)

Human skin keratinocytes (KCs) - Passage 3

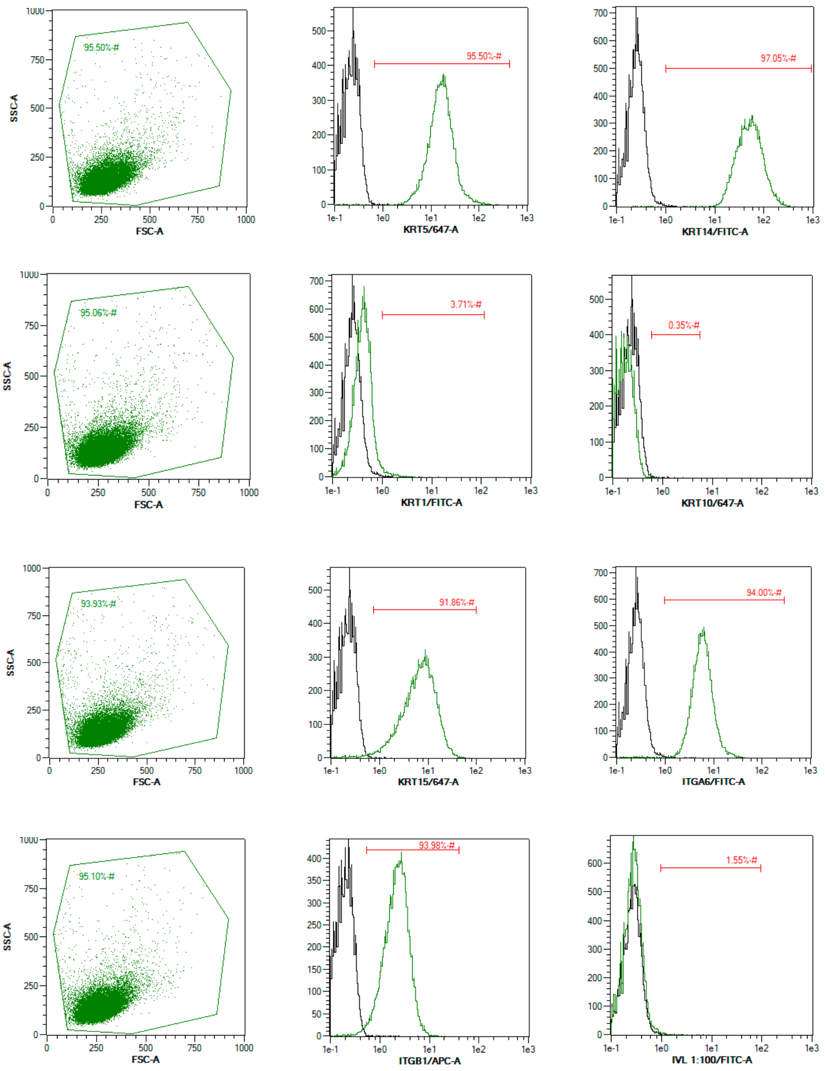

Supplementary Figure S1(a). Representative flow cytometric analyses of skin keratinocytes stained with various known basal-, differentiated- and cell adhesion markers for human epithelial cells

b) Human umbilical cord lining epithelial cells (CLECs) – Passage 3

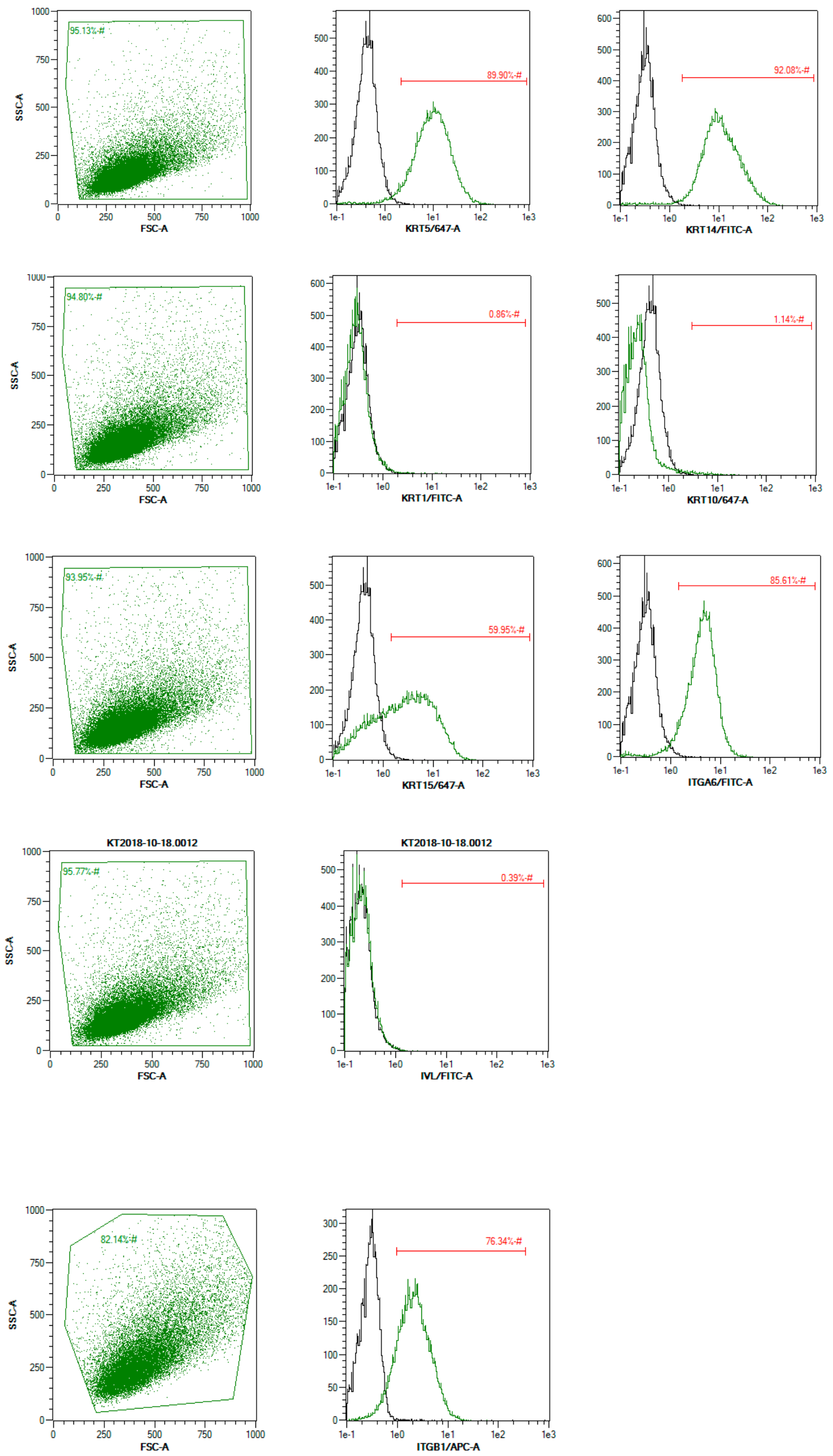

Supplementary Figure S1(b). Representative flow cytometric analyses of umbilical cord lining epithelial cells stained with various known basal-, differentiated- and cell adhesion markers for human epithelial cells

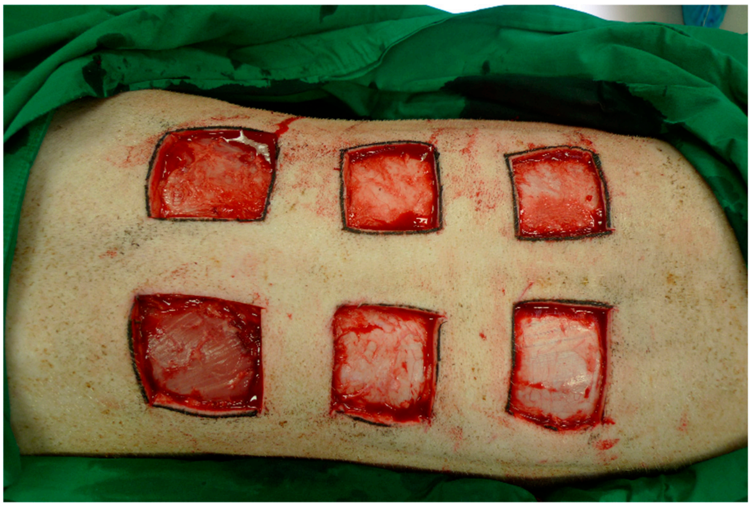

Supplementary Figure S2: Porcine full thickness excisional wound model with 6 full thickness excisional wounds (5cm by 5cm) created on the dorsal area of each Yorkshire pig.

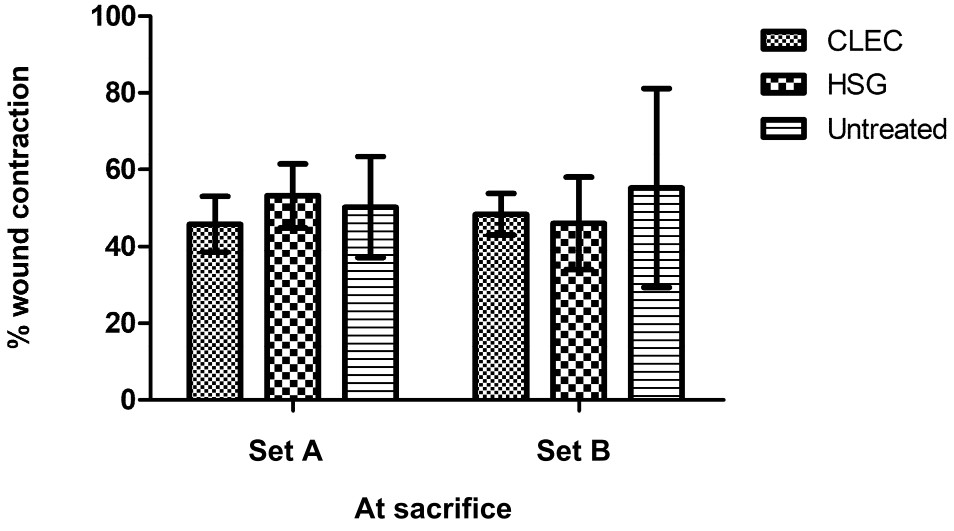

Supplementary Figure S3: Percentage wound contraction represented by mean ± SD based on the tattooed area boundaries (when the animal was euthanised) with respect to the original tattooed area during wound creation on the porcine model. The three treatment arms compared were CLEC, HSG and standard dressings ("untreated"). Statistical analysis was performed using one-way ANOVA – Bonferroni's Multiple Comparison Test and there was no significant difference between each treatment arm for the two sets studied.

Supplementary Table S1. Tissues and tissue-derived cells used for the experiments

Keratinocytes from foreskin

Donor ID	Age	Gender
KC #009	6	M
KC #015	3	M
KC #020	6	M
KC #021	7	M

Epithelial cells from umbilical cord tissue lining

Donor ID	Gender
CLEC #030	M
CLEC #031	F
CLEC #032	F
CLEC #044	M

De-identified skin grafts and dermal fibroblasts from the Singapore General Hospital Skin Bank Unit

Tissue ID	Specimen Type
#11002	Cryopreserved human skin allografts
#NFTST09	Cryopreserved dermal skin fibroblasts

Supplementary Table S2. List of antibodies used in this study, related to Figure 1, Supplementary Figure S1 and Figures 2b-c.

Antibodies	Source	Catalog number	Dilution
Alexa-Fluor 647- conjugated KRT5	Abcam	ab193895	1:400 IF; 1:200 FC
FITC- conjugated KRT14(clone LL002)	Abcam	ab77684	1:100 IF; 1:100 FC
KRT1 (clone LHK1)	Abcam	ab81623	1:100 IF; 1:200 FC
Alexa- Fluor 647 conjugated KRT10	Abcam	Ab194231	1:100 IF; 1:100 FC
KRT15 (Clone EPR1614Y)	Abcam	Ab52816	1:100 IF; 1:50 FC
IVL(clone SY5)	Abcam	Ab68	1:50 IF only
IVL (SY5)	Novus Biologicals	NBP2-34264	1:100 FC only
FITC-conjugated CD49f (*ITGA6*)	BD Pharmingen	555735	1:20 IF; 1:50 FC
APC-conjugated CD29 (*ITGB1*)	BD Pharmingen	559883	1:20 IF; 1:50 FC
p63 (Clone 4A4)	Santa Cruz	Sc-8431	1:20 IF; 1:100 FC
Mouse IgG2a,monoclonal (FITC) Isotype control	Abcam	Ab81197	1:100
Alexa-Fluor 488- conjugated goat anti-rabbit	Life Technologies	A11008	1:1000
Alexa- Fluor 488-conjugated goat anti-mouse	Life Technologies	A11001	1:1000
Alexa-Fluor 647- conjugated goat anti-rabbit	Life Technologies	A21245	1:1000

IF: Immunofluorescence; FC: Flow cytometry
